# Supplementary material for: A genomic analysis and transcriptomic atlas of gene expression in Psoroptes ovis reveals feeding- and stage-specific patterns of allergen expression
Source: BMC Genomics. 2019 Oct 23;20:756. doi: 10.1186/s12864-019-6082-6 (PMC6806590; doi:10.1186/s12864-019-6082-6)
Supplement: Supplementary file 1 — Additional file 1: Table S1. Total number of Illumina Solexa Hi-Seq reads and the percentage of reads pseudo-mapped for each sample to the P. ovis transcriptome. Data shown for each of the fifteen RNA samples from P. ovis life-cycle stages and for “fed” (F) and “starved” (S) mites. AF = adult females, AM = adult males, L = larvae, P = protonymph, T = tritonymph. [file 12864_2019_6082_MOESM1_ESM.docx]

| **Sample Description** | **Sample ID** | **Total Reads** | **Percentage mapped reads** |
| --- | --- | --- | --- |
| Adult Females | AF_1 | 12,660,138 | 85.3 |
| Adult Females | AF_2 | 16,172,943 | 85 |
| Adult Females | AF_3 | 15,960,093 | 84.3 |
| Adult Males | AM_1 | 13,822,039 | 81.1 |
| Adult Males | AM_2 | 17,998,180 | 82.3 |
| Adult Males | AM_3 | 9,886,599 | 79.1 |
| Larvae | L_1 | 14,670,039 | 82.1 |
| Larvae | L_2 | 12,066,561 | 82.2 |
| Larvae | L_3 | 13,094,220 | 84.1 |
| Protonymphs | P_1 | 11,038,552 | 76.3 |
| Protonymphs | P_2 | 19,996,884 | 76.4 |
| Protonymphs | P_3 | 13,850,774 | 75.9 |
| Tritonymphs | T_1 | 26,140,689 | 83.5 |
| Tritonymphs | T_2 | 10,155,712 | 84.6 |
| Tritonymphs | T_3 | 8,524,992 | 85.1 |
| “Fed” | F_1 | 9,454,291 | 89 |
| “Fed” | F_2 | 16,091,427 | 86.6 |
| “Fed” | F_3 | 12,419,967 | 88.3 |
| “Starved” | S_1 | 12,851,430 | 83.9 |
| “Starved” | S_2 | 13,229,126 | 83.2 |
| “Starved” | S_3 | 8,183,501 | 82.9 |

**Table S1.** Total number of Illumina Solexa Hi-Seq reads. Data shown for each of the fifteen RNA samples from *P. ovis* life-cycle stages and for “fed” (F) and “starved” (S) mites. AF = adult females, AM = adult males, L = larvae, P = protonymph, T = tritonymph.
